# Comparison of the COVID-2019 (SARS-CoV-2) pathogenesis with SARS-CoV and MERS-CoV infections

**DOI:** 10.2217/fvl-2020-0050

**Published:** 2020-05-20

**Authors:** Mona Fani, Ali Teimoori, Shokouh Ghafari

**Affiliations:** 1 ^1^Virology Department, School of Medicine, Ahvaz Jundishapur University of Medical Sciences, Ahvaz, Iran; 2 ^2^Department of Virology, Faculty of Medicine, Hamadan University of Medical Sciences, Hamadan, Iran; 3 ^3^Infectious Diseases Research Center, Birjand University of Medical Sciences, Birjand, Iran

**Keywords:** ACE2, coronavirus, COVID-19, SARS-CoV-2, zoonotic diseases

## Abstract

The severe acute respiratory syndrome coronavirus 2 (SARS-CoV-2), was first identified in several patients who traveled to Wuhan or went to a seafood wholesale market in Wuhan. The phylogenetic tree showed that SARS-CoV-2 was 96.2% identical to bat β-coronaviruses from lineage B. Also, several studies reported that SARS-CoV-2 uses the SARS-CoV receptor, angiotensin-converting enzyme 2, for entry to target cells. Lung alveolar and small intestine are potential targets for SARS-CoV-2 due to the high expression of the angiotensin-converting enzyme 2 receptor. In this review, we focused on the zoonotic β-coronaviruses and given there is no specific drug or vaccine for coronavirus disease 2019, we reviewed the literature on the therapy options for SARS and Middle East respiratory syndrome coronavirus infection, in order to discover their possible use in the treatment of SARS-CoV-2 infections.

Although several coronaviruses are known as the major causes of morbidity and mortality in animals, their importance was highlighted after the emergence of SARS (severe acute respiratory syndrome). Furthermore, some studies indicated a coronavirus interspecies transmission from animals to human that is significant for global health institutions. Generally, coronavirus has a wide host range including birds, felines, pigs, cows, turkeys and dogs; causes respiratory, enteric, hepatic and neurologic infections. Coronaviruses cause mild-to-severe disease in humans and they have newly emerged from a zoonotic source. On the other hand, nowadays it is believed that approximately 75% of infectious diseases are zoonotic. Documented evidence indicates the mutation of existing strains, leading to the emergence of novel strains and new illnesses in animals [1].

Coronavirus belongs to the family *Coronaviridae* and the subfamily *Coronavirinae* and based on genetic properties, this subfamily has been divided into four genera: α-coronavirus, β-coronavirus, γ-coronavirus and δ-coronavirus [2].

In the past two decades, β-coronavirus has been a major subject of research due to it emerging and re-emerging. Human coronavirus (HCoV) infects the upper and lower respiratory tract in children, aged people and patients with underlying heart and respiratory diseases [3].

HCoV is a positive-sense RNA virus and has the largest genome known among RNA viruses. Also, 229E, OC43, NL63, HKU1, SARS, MERS (Middle East respiratory syndrome) and coronavirus disease 2019 (COVID-19; SARS-CoV-2) species cause respiratory tract infection. Among them, 229E, OC43, NL63 and HKU1 strains result in common cold symptoms in individuals. The two other species, SARS-CoV and MERS-CoV which belong to β-coronavirus genus sometimes are associated with fatal disease. Recently, the SARS-CoV-2 strain was reported by the Chinese Center for Disease Control and Prevention (China CDC) in Wuhan city on 31 December 2019 [4].

Structural proteins are essential for the assembly and infection of coronavirus: spike glycoprotein (S) on the surface of the particle consists of S1 and S2 subunits. The S1 subunit contains the receptor binding domain (RBD) and binds to the cellular receptor and the S2 subunit facilitates the fusion and entrance process. Membrane (M) protein by increasing the membrane curvature, promotes the viral assembly. Envelope (E) protein is essential to release the virus. Nucleocapsid (N) protein is interferon (IFN) antagonistic and supports viral replication. The nonstructural proteins of coronaviruses can block the host immune system for viral replication [4].

RNA-dependent RNA polymerase (RdRp) enzyme in coronaviruses has proofreading-activity, so the mutation rate in this family is lower than other RNA viruses, while homologous recombination frequently occurs in this family [5].

In this review, we compared the pathogenesis of SARS-CoV-2 infection with SARS-CoV and MERS-CoV infections and briefly mentioned the symptoms and transmission pathway of COVID-19. We also introduced the potential targets for therapeutic options to treat COVID-19.

## Etiology of severe acute respiratory syndrome coronavirus infection

SARS-CoV was a pandemic agent of the SARS from 2002 to 2003 in 33 countries with 8096 cases and 774 deaths [6]. In 2003, Holmes reported that the sudden emergence of SARS-CoV did not correlate to mutation or recombination between previous HCoV. On the other hand, genome sequencing and epidemiologic reports demonstrated that SARS-CoV was a new virus which was not similar to known HCoV [7]. However, the genome sequences of human SARS-CoV were similar to animal isolates and in addition, several serological studies confirmed that animal traders had specific antibody (IgG) against the SARS-CoV infection. These results displayed that SARS-CoV was a zoonotic virus and originated from animal and bird species before outbreaks in humans [1]. Moreover, in 2006, Li *et al*. reported that significant genetic changes occurred in the spike glycoprotein (S glycoprotein) of bat SARS-CoV to infect humans. Finally, the sequence data of SARS-CoV exhibited 87–92% identity with bat SARS-CoV and it was concluded bats were the potential natural reservoir for the outbreak of SARS in 2003 [8]. In fact, exotic animals have transmitted SARS-CoV to humans through intermediate hosts (civet cats and raccoon dogs) and subsequently, person-to-person transmission resulted in the outbreak of SARS-CoV in hotels and hospitals [9].

Several risk factors including age, diabetes and heart disease can increase the risk of death. SARS can infect the respiratory tract of individuals in all age groups, principally through droplet transmission. SARS-CoV infection is associated with several common signs such as fever, diarrhea, myalgia, malaise and chills [9].

The entry of SARS-CoV is facilitated by attachment of S glycoprotein to ACE2, subsequently, the conformational changes of S glycoprotein take place in the endosome microenvironment by cellular serine protease cathepsins B and L to facilitate the fusion process [9].

In 2005, Li *et al*. reported that residues 318–510 of the S1 domain encode the RBD, but two of amino acids are not conserved in SARS-CoV strains. Probably, the adaptation of S glycoprotein with ACE2 permits the efficient infection of human cells and also cause the unusual severity of SARS-CoV [10].

The ACE2 is expressed on epithelial cells of the lung, tongue, kidney, heart and liver. The attachment of S glycoprotein to ACE2 can cause the loss of cilia, squamous metaplasia and an increase in macrophages in the alveoli that cause diffuse alveolar damage to the lung [11].

Furthermore, SARS-CoV produces 3a and 7a proteins that cause apoptosis in the lungs, kidneys and liver cells. Also, activation of TH1 and increasing inflammatory cytokines and interleukins such as IFN-γ -IP-10, IFN-γ, IL-1B, IL-6, IL-8, IL-12 and MCP-1 happen in SARS-CoV infection [12].

## Etiology of MERS-CoV infection

In 2012, a new human disease that was caused by MERS-CoV emerged in the Middle East with 2494 confirmed cases and 858 fatalities [13,14]. For a long time, the origin of MERS-CoV was controversial, previously it was thought that bat was the reservoir due to phylogenetic similarity of MERS-CoV with certain bat coronaviruses. But serological and phylogenetic studies demonstrated that dromedary camels suffer from human-MERS-CoV-like disease. After camel–human contact, human-to-human transmission occurred, especially in healthcare communities [13].

The average incubation period for MERS-CoV is 5–7 days but can be as long as 2–14 days. Also, MERS-CoV infects men more than women. The clinical symptoms of MERS may be asymptomatic, mild and can lead to severe disease with multi-organ failure. Also, MERS is associated with metabolic syndromes including diabetes mellitus, cardiovascular diseases and obesity. Subsequently, metabolic syndrome can interfere with innate and humoral immune and can render patients more susceptible to infectious diseases.

DPP4, CD26 is the receptor for attachment of MERS-CoV to the pneumocytes and epithelial cells of the respiratory tract [15]. Moreover, MERS‐CoV has a specific RBD that is 231-amino-acid in S glycoprotein and binds to DDP4 on host target cells. DPP4 affects glucose metabolism, T cell activation, cytotoxic modulation, cell adhesion and apoptosis. MERS-CoV RBD comprises the core structure and a RBM. Although the core structures of MERS CoV and SARS-CoV RBDs are highly similar, their RBMs are divergent and lead to different receptor specificities. Also, it was supposed that MERS-CoV transmission from bats-to-humans and then from human-to-human occurred through little or no adaptation in RBD [16].

## Etiology of SARS-CoV-2 infection

The new coronavirus was named SARS-CoV-2, belongs to β-coronaviruses based on the genome sequence and infects the upper and lower respiratory tract. Symptoms of the novel coronavirus strain are milder than SARS and MERS, but it transmits from human-to-human faster than them. Besides, the mortality rate of SARS-CoV-2 is lower (3.4%) than that of SARS-CoV (9.6%) and MERS (35%) [5].

More recent studies, have confirmed that diabetes and hypertension may relate to the pathogenesis of SARS-CoV-2. By blocking the function of lymphocytes and macrophages, these disorders reduce IFN-γ and interleukin synthesis to downregulate the host innate immune response [2,12].

Despite SARS-CoV-2 mostly affecting the middle-aged and older people with underlying disease, it does not mean that children are less susceptible to novel coronaviruses. Maybe their relative resistance to SARS-CoV-2 infection may be due to the active immune system and the healthy respiratory tract compared with adults. Laboratory mice models showed that the ACE2 expression as the receptor of the SARS-CoV-2 decreases with age. Although this result contradicts with low susceptibility of children to SARS-CoV-2 infection, the lung is protected by ACE2 against SARS, influenza A H5N1 virus, respiratory syncytial virus infections. It can be explained that ACE2 in healthy people and children modulates the renin–angiotensin system via cleaving angiotensin (Ang)-II to Ang-1–7 to prevent severe acute lung failure. Indeed, the severe lung injury arising from a viral respiratory infection is associated with ACE2 deficiency and increasing of Ang II [17,18].

Zhou *et al.* demonstrated that Asian men are more susceptible to SARS-CoV-2 infection compared with women and other races due to more expression of the ACE2 receptor [19].

According to the latest studies, SARS-CoV-2 has the highest number of casualties in more than 80 countries and is now a pandemic.

The incubation period and the epidemiological, clinical, laboratory and radiological features of patients with confirmed COVID-19 were similar to SARS-infected people in 2003, but phylogenetic tree analysis showed that the SARS-CoV-2 is separate from SARS and MERS. On the other hand, the outbreak of SARS-CoV-2 has probably started from the wholesale market of Huanan seafood, where wildlife such as snakes, bats, birds, frogs, hedgehogs and rabbits are sold. Wei Ji *et al*. using sequence analysis of different species of coronavirus revealed that SARS-CoV-2 is a recombinant virus between the bat coronavirus and a source-unknown coronavirus, but the possible intermediate host of SARS-CoV-2 is the pangolin [11]. So, these results indicate the outbreak in Wuhan city was a zoonosis disease similar to bat SARS [2,12]. Moreover, the viral genome sequencing confirmed that SARS-CoV-2 is 96.2% identical to some bat coronaviruses and also more distantly correlated to SARS-CoV and MERS-CoV (about 79 and 50%, respectively). Also, the genome sequencing of SARS-CoV-2 S2 protein confirmed the similarity of 93% with bat coronaviruses.

Moreover, the SARS-CoV-2 and SARS-CoV are distinct from each other in the genome sequence of RNA-dependent RNA polymerase (RdRp). Therefore SARS-COV-2 was clustered within an independent subclade in the β-coronavirus genus [14].

The phylogenetic analysis of RBD showed that the SARS-CoV-2 was close to SARS-CoV that was located in lineage B, also, SARS-CoV-2 can infect BHK-21 cells. These results suggest that SARS-CoV-2 uses the ACE2 as a cell receptor and cellular proteases like TMPRSS2 protease for SARS-COV-2 S glycoprotein priming [16].

Lu *et al*. observed that several residues in RBD of SARS-CoV S glycoprotein were variable in SARS-CoV-2 [14]. Also, biophysical and cryo-EM structure evidence revealed the affinity of SARS-CoV-2 S protein to ACE2 is 10–20-times higher than the SARS-CoV one. These findings support the theory of the higher contagion of SARS-CoV-2 compared with SARS-CoV [11,20].

Indeed, the ACE2 is a physiologically related receptor during coronavirus infections and responsible for the localization of viruses in infected human and animals. Therefore, the infection efficiency correlates with the ability of the ACE2 of each species to support viral replication. It should be noted that several bat SARS-CoVs did not employ ACE2 for entry to the target cell. The analysis reports showed that the most amino acids in RBM of S glycoprotein of SARS-CoV-2 were similar to bat SARS-CoV in lineage B which uses ACE2 on the target cell surface [16].

## Diagnosis of SARS-CoV-2 infection

Fever, cough and fatigue are common symptoms of COVID-19. Also, muscle ache, chest pain, dyspnea, sore throat, vomiting, diarrhea and confusion are observed in SARS-CoV-2 infection. Along with clinical symptoms, the C-reactive protein and cytokine level increases and the total white blood cell, lymphocyte, platelet and thromboplastin time decrease. Acute respiratory distress syndrome is a common complication in patients, followed by anemia, acute heart damage and secondary infections. Unilateral and bilateral pneumonia is found in the chest computed tomography (CT) images or chest x-ray in the patients with COVID-19.

Real-time reverse transcriptase-PCR of nasopharyngeal swab is routinely used to detect SARS-CoV-2 (Table 1).

**Table 1.** Detection methods and genes for diagnosis of severe acute respiratory syndrome coronavirus 2.

Sample	Method	Gene	Primers & probe (5′-3′)	Comments	Ref.
Throat swab sample	rRT-PCR	ORF1ab	(F)primer: CCCTGTGGGTTTTACACTTAA (R)primer: ACGATTGTGCATCAGCTGA Probe: VIC-CCGTCTGCGGTATGTGGAAAGGTTATGG-BHQ1	Respiratory tract specimens were used to diagnose NCIP through RT-PCR. The serum of patients was not obtained to evaluate the viremia. The viral load is a potentially useful marker associated with disease severity of coronavirus infection and this should be determined in NCIP.	[21]
		N gene	(F)primer: GGGGAACTTCTCCTGCTAGAAT (R)primer: CAGACATTTTGCTCTCAAGCTG Probe: FAM- TTGCTGCTGCTTGACAGATT-TAMRA		
Blood, sputum, feces, urine and nasal samples	rRT-PCR	ORF1ab	(F)primer: CCCTGTGGGTTTTACACTTAA (R)primer: ACGATTGTGCATCAGCTGA Probe: VIC-CCGTCTGCGGTATGTGGAAAGGTTATGG-BHQ1	1. Lower respiratory tract samples were positive. 2. Live virus was detected in feces	[22]
Oral swabs, anal swabs and blood samples throat swabs, sputum, urine and stool	qRT-PCR assay	S gene	(F)primer: CAATGGTTTAACAGGCACAGG (R)primer: CTCAAGTGTCTGTGGATCACG Probe: NM	The virus may be present in anal swabs or blood of patients when oral swabs detection negative. Patients infected with SARS-CoV-2 may harbor the virus in the intestine at the early or late stage of disease.	[19,23]
Throat swabs, sputum, urine and stool	qRT-PCR assay	N gene	Primers: NM Probe: NM	The peaks of viral loads in throat swab and sputum samples were 5–6 days after symptom onset, ranging from around 10^4^ to 10^7^ copies per ml during. Sputum samples generally showed higher viral loads than throat swab samples.	[24]
Nasopharyngeal and throat swabs and stool and urine samples	Real-timeRT-PCR	S gene	(F)primer: CCTACTAAATTAAATGATCTCTGCTTTACT (R)primer: CAAGCTATAACGCAGCCTGTA		[2]
	Multiplex RT-PCR	RdRp	(F)primer: CAAGTGGGGTAAGGCTAGACTTT (R)primer: ACTTAGGATAATCCCAACCCAT		
Nasal and pharyngeal swabs, bronchoalveolar lavage fluid, sputum or bronchial aspirates	NGS & real-time RT-PCR	E	(F)primer: TCAGAATGCCAATCTCCCCAAC (R)primer: AAAGGTCCACCCGATACATTGA Probe: CY5-CTAGTTACACTAGCCATCCTTACTGC-BHQ1		[12]
Blood, stool and urine samples and nasopharyngeal swabs	PCR	RdRp	NM	Virus was detected by PCR in 50% stool, 8% in whole blood and virus was not detected in urine	[25]

NM: Not mentioned; NCIP: Novel coronavirus (2019-nCoV)-infected pneumonia; rRT-PCR: Real-time reverse transcriptase-PCR; SARS-CoV-2: Severe acute respiratory syndrome coronavirus 2.

Because chest CT is more sensitive than real-time reverse transcriptase-PCR, the combination of SARS-CoV-2 molecular tests and clinical features is used to diagnose COVID-19 [26].

## Therapeutic options for SARS-CoV-2 infection

The SARS-CoV-2 infection appears to be out of control, so drugs and vaccines will be needed to prevent public health threats.

To date, no licensed vaccines or proven therapies exist against SARS-CoV-2, but the combination of IFNs and ribavirin is effective for coronaviruses infection. Ribavirin targets viral replication to block the viral RNA synthesis and mRNA capping [11].

Individuals with COVID-19 have high amounts of IL-1B, IFNγ, IP10 and MCP1 that can lead to activated Th1 cell responses. On the other hand, in contrast to SARS-CoV infection, secretion of IL-4 and IL-10 has been reported in COVID-19, that suppress inflammation, which could be one of the reasons for the lower severity of COVID-19 compared with SARS infection [12]. Vaccination can provide the best line of defense against disease compared with chemotherapeutic drugs. Generally, more research is required on Th1 and Th2 responses against SARS-CoV-2 to clarify the pathogenesis.

In a study, it was demonstrated that the serum from a patient with SARS-CoV S glycoprotein prevent the entry of SARS-CoV-2 [27]. More broadly, *in vitro* researches are needed to determine the inhibitory effect of SARS-CoV-infected serum on replication of SARS-CoV-2.

Lopinavir and ritonavir are anti-CoV drugs that target the nonstructural proteins of chymotrypsin-like protease (3CLpro) and polymerases, however, none of them are licensed for clinical trials yet [28].

S glycoprotein and ACE2 are critical in SARS-CoV-2 infection, thus, employing them can help to develop antiviral agents. Chloroquine is a potent drug against SARS-CoV-2 infection that increases endosomal pH and also blocks the cathepsin function, moreover, chloroquine can interfere with the virus cell binding [16]. Therefore, TMPRSS2 may be as a suitable therapeutic option, because TMPRSS2 in SARS-CoV-2, like SARS-CoV, help to spread SARS-CoV-2 via virus/cell to cell fusion and also by diminishing viral identification by neutralizing antibodies. Thus, using a protease inhibitor such as camostat mesylate could block the TMPRSS2 function.

Remdesivir and favipiravir target the RdRp enzyme and lead to premature termination during virus transcription, therefore, they can be used in the treatment of COVID-19. But further studies on the effect of chloroquine, remdesivir and favipiravir on extracellular proteases are required in an *in vivo* setting. Furthermore, several anti-HIV drugs including darunavir, cobicistat and ASC09F have been considered for the clinical trials against SARS-CoV-2 infection [11].

In 2003, Kumar *et al.* reported that ACE2 in the form of nucleic acid shuttles can treat acute respiratory and lung failure arising from SARS-CoV infections [27]. Also, in 2016, Gu *et al*. published a paper in which they demonstrated that a recombinant ACE2 reduces the lung injury and regulates the innate immune system [18]. Moreover, in 2018, Guangyu found that nanobodies (single-domain antibodies) can target the MERS-CoV RBD to inhibit the binding of S glycoprotein to DPP4 [29]. Thus, ACE2 receptor and RBD can be other therapeutic choices.

## Conclusion

Bat seems the common natural origin of SARS-CoV and MERS-CoV and SARS-CoV-2. The clinical features of them are similar and unlike SARS-CoV and MERS-CoV, SARS-CoV-2 spreads rapidly. On the other hand, the adaptation of the S glycoprotein and its affinity for ACE2 can determine the severity of SARS-CoV-2 infection. Thus, a vaccine containing S glycoprotein and inactivated SARS-CoV-2 could have the potential to prevent COVID-19. What is now needed is research on recombination events and genetic diversity of SARS-CoV-2 to present an effective vaccine or drug.

## Future perspective

There are concerns about the emergence of large numbers of people infected with SARS-CoV-2 in the short term, which could lead to an increase in the mortality rate. As the number of patients increase, the process of controlling the disease is further disrupted in the healthcare system, which is more detrimental to individuals with a history of immunodeficiency. To date, there are no effective vaccines and drugs against COVID-19. By reviewing the literature on the therapeutic options for people infected with SARS and MERS-CoV, we introduced therapeutic options for SARS-CoV-2 infection. However, further clinical studies would clarify the value of our findings.

**Summary points**

- Severe acute respiratory syndrome coronavirus 2 (SARS-CoV-2) infects the upper and lower respiratory tract.
- SARS-CoV-2 Spike (S) protein had higher affinity to human angiotensin-converting enzyme 2 receptor than that of SARS-CoV.
- COVID-19 is a zoonotic disease similar to bat SARS infection.
- Viral genome sequencing confirmed that SARS-CoV-2 is 96.2% identical to some bat coronaviruses.
- Fever, cough and fatigue are common symptoms of COVID-19.
- Real-time reverse transcriptase-PCR of nasopharyngeal swab is routinely used to detect the SARS-CoV-2.
- Chloroquine and remdesivir showed the most powerful antiviral activities against SARS-CoV-2 infection.
